# Unravelling the transmission dynamics and evolutionary trajectory of mpox in South Korea: 2022–2025 outbreak

**DOI:** 10.1080/22221751.2026.2746255

**Published:** 2026-10-07

**Authors:** Minji Lee, Chi-Hwan Choi, Sang Eun Lee, Jin-Won Kim, Gyuri Sim, Jeong Hyun Lee, Hwajung Yi, Yoon-Seok Chung

**Affiliations:** Division of High-risk Pathogens, Department of Laboratory Diagnosis and Analysis, Korea Disease Control and Prevention Agency, Cheongju, Republic of Korea

**Keywords:** Mpox, Monkeypox virus, APOBEC3-mediated mutagenesis, genomic surveillance, molecular evolution, transmission dynamics, next-generation sequencing, viral adaptation

## Abstract

The 2022 monkeypox (mpox) outbreak, driven by clade IIb monkeypox virus (MPXV), was facilitated by human-to-human transmission, particularly within heavy-tailed sexual networks. APOBEC3-associated mutations have been a prominent feature of MPXV genomic evolution during the global outbreak. National genomic data were collected from clinical samples by the Korea Disease Control and Prevention Agency to examine the transmission dynamics and molecular evolution of MPXV. Target enrichment-based next-generation sequencing was used to generate high-quality genomes. Mutation profiling was used to identify key genetic changes; clustering based on common variants provided insights into national transmission dynamics and molecular evolution. Analysis of mpox-positive samples revealed the linkage of most cases to sub-lineage C.1 and a few cases to C.1.1. Genomic clustering revealed an uneven distribution of cases, with a dominant cluster accounting for a substantial proportion of domestic transmission while most other cluster remained relatively limited in size, consistent with heterogeneous transmission patterns. APOBEC3-associated mutations progressively accumulated during the outbreak and were frequently observed in genomic regions associated with viral structural integrity and cell-to-cell transmission and host-modulation, indicating continued genomic diversification during human-to-human transmission. Together with the dominance of localized clusters, these findings suggest heterogeneous transmission patterns and the possible contribution of epidemiologically unrecognized transmission to the spread of MPXV in South Korea. These results underscore value of integrating genomic surveillance and targeted interventions to characterize transmission patterns and monitor viral genomic changes of potential biological or public health relevance.

## Introduction

Monkeypox (mpox), a disease endemic to Central and West Africa, is caused by the clade I monkeypox virus (MPXV). Infections occur through contact with animals, via both animal-to-animal and animal-to-human transmission. The case fatality rates (CFRs) associated with clade I MPXV range from 1·4% to 10% [1]. Infections in the United States that occurred in 2003 were caused by animals imported from Africa, resulting in a multistate outbreak in the Midwest. The 2003 outbreak, similar to that in the endemic regions, involved animal-to-human transmission and was caused by clade IIa MPXV [2]. Following the 2003 outbreak, no global outbreaks were reported until 2022, when an infection occurred in an individual who had travelled to Nigeria. The virus spread to multiple contacts, predominantly affecting a specific sexual network. This was followed by a marked increase in human-to-human transmission, particularly through unprotected sexual contact, culminating in a global outbreak [3,4]. The 2022 global outbreak was driven by clade IIb MPXV, which accumulates single-nucleotide polymorphisms (SNPs) despite being a relatively large and mutation-stable DNA virus. This increase in SNP frequency is closely linked to apolipoprotein B mRNA editing catalytic polypeptide-like 3 (APOBEC3)-mediated editing, characterized by TC-to-TT and GA-to-AA mutations and likely sustained by human-to-human transmission [5]. The range of reported CFRs associated with clade IIb MPXV is 0·1–3·6%, relatively intermediate compared to that associated with clade I MPXV [1,6]. Since 2022, mpox has spread globally, including sustained transmission in multiple non-endemic regions [7]. During 2023–2024, the Democratic Republic of the Congo (DRC) experienced an increase in suspected cases. These cases were confirmed infections with clade I MPXV, exhibiting greater disease severity and higher fatality rates than those caused by the MPXV IIb strain responsible for the 2022 outbreak. Notably, this clade was classified as clade Ib, genetically distinct from the previously known clade I strains. Given the 2022 epidemic, this clade represents a major threat to global public health and has received global attention. Furthermore, among recently reported cases of mpox outbreaks in endemic regions, transmission has transitioned from animal-to-human to primarily human-to-human, mediated through close contact [8,9]. Amid several historical outbreaks and successive PHEIC declarations [10,11], mpox remains continues to circulate, with sporadic transmission persisting worldwide. Localized epidemics and the threat of global spread necessitate proactive genomic surveillance, providing rich genomic information for interpreting epidemic dynamics and viral evolution. In this study, we aimed to characterize the genomic transmission patterns and molecular evolution of MPXV during sustained human-to-human transmission in South Korea using national genomic surveillance data. Our study contributes to mitigating the imbalance in genomic data availability [12] by providing comprehensive genomic insights, thereby offering rational perspectives on the evolutionary and epidemiological processes of MPXV.

## Methods

### Experimental specimens and ethical approval

This study was conducted as an integral part of national public health efforts led by the Korea Disease Control and Prevention Agency, aimed at advancing disease control strategies and infectious disease management. A total of 166 residual clinical specimens from laboratory-confirmed mpox cases were analyzed. All available residual specimens provided to our laboratory were included in the analysis without additional selection criteria. No personally identifiable information was gathered, and all procedures were conducted in compliance with established ethics guidelines. Consequently, formal institutional review board approval was not deemed necessary.

### Genome sequencing and phylogenetic analysis

Depending on the sample characteristics and sequencing strategy, high-quality consensus genome sequences were generated using one of two distinct workflows. In the primary hybrid-capture approach, DNA libraries were prepared utilizing the Illumina Viral Surveillance Panel (or alternatively, the Qiagen MPXV panel), and whole-genome sequencing was performed on the Illumina Miseq platform (2 × 150-bp format) using the MiSeq Reagent Kit v2. Raw paired-end reads were processed using CLC Genomics Workbench 26 (CGW26). Following the removal of adapter sequences, reads with quality scores below Q30 were trimmed at a threshold of 0.001. To deplete host-derived sequences, reads were mapped against the human reference genome (GRCh38.p14). The remaining unmapped reads were subsampled to 1,000,000 reads and aligned to the Mpox reference genome (GenBank accession number: NC_063383) using a length fraction of 0.5 and similarity fraction 0.8, followed by local realignment and the extraction of consensus sequences. These extracted sequences were then manually curated to finalize complete and near-complete genomes. Alternatively, in the amplicon-based approach, DNA libraries were prepared using the Thermo Fisher MPXV AmpliSeq panel and sequenced on the Thermo Fisher Genexus System, generating single-end reads. The resulting data were directly processed to extract consensus sequences utilizing the proprietary Thermo Fisher pipeline, without further manual curation. All resulting genomes from both workflows were assigned to respective clades and lineage using Nextcalde v2.14.0. For phylogenetic inference, these genomes were first aligned (augur align), and maximum-likelihood phylogenies were subsequently reconstructed from these aligned sequences using IQ-TREE (augur tree) integrated within the Nextstrain CLI v7.2.0 pipeline. Within this framework, time-resolved phylogenies were generated by temporally refining the trees using the TreeTime implementation (augur refine), with laboratory confirmation dates serving as sampling dates. Branch-defining mutations identified via the Nextstrain pipeline were independently verified through variant calling using Snippy, incorporating FreeBayes for variant detection. High-confidence variants were strictly filtered applying Snippy’s default thresholds (minimum coverage depth ≥ 10×, variant allele frequency ≥ 0.90, and Phred-scaled quality score ≥ 100). To investigate APOBEC3-mediated mutational signatures, these validated variants were analyzed using the mutation_profile tool (https://github.com/insapathogenomics/mutation_profile). Finally, the resulting phylogenetic trees and corresponding mutation patterns were visualized using SNIPIT (Appendix p 1).

### Phylogenetic definition of genomic subcluster

Genomic subclusters were defined primarily based on the phylogenetic topology inferred using the Nextstrain CLI v7.2.0 pipeline, without applying a fixed SNP-distance threshold, bootstrap-support cutoff, or epidemiological information during the initial clustering phase. Instead, branch-defining mutations identified within the Nextstrain framework, while carefully accounting for homoplasy, were utilized to support subcluster assignments. Monophyletic groups generally comprising five or more cases were operationally designated as genomic subclusters for downstream analyses. This five-case threshold served as a practical heuristic rather than a strict limit; phylogenetically distinct groups comprising fewer cases were also retained as independent subclusters when strongly supported by the tree topology and characteristic branch-defining mutations. As described above, these mutations were independently validated via Snippy, annotated for predicted functional effects, and visualized using SNIPIT to confirm the unique mutation profile of each subcluster. Finally, epidemiological information was overlaid only after the genomic subclusters were fully defined, ensuring an unbiased assessment of the concordance between the genomic clustering and observed transmission patterns.

### Molecular evolution analysis

Low-frequency variants were independently identified using mutation-profile and Snippy analyses based on the clade IIb reference genome (NC_063383) to characterize mutation burden and functional variation across patient-derived sequences and approximately 9,800 publicly available MPXV genomes. Variants detected by either approach were combined into a non-redundant set and mapped to their corresponding coding sequences. Pairwise dN/dS ratios were subsequently estimated with KaKs Calculator, using the clade I reference genome (KJ642616) as an evolutionarily divergent reference. Given the high genomic stability and limited natural variation characteristic of this double-stranded DNA virus, this distant reference was utilized as a broad comparative baseline to provide an exploratory assessment of the evolutionary characteristics of mutation-containing genes. Genes exhibiting elevated pairwise dN/dS ratios (≥1) were further interpreted together with their mutation profiles and annotated biological functions.

### Statistical analysis

Genomic cluster-size distributions were evaluated by fitting Poisson and negative binomial models. A Poisson model was initially fitted as the baseline count model, followed by a negative binomial model to account for overdispersion. Overdispersion was assessed using the variance-to-mean ratio (VMR), Pearson dispersion statistic, and the estimated dispersion parameter (θ). Model fit was compared using the Akaike Information Criterion (AIC) and Bayesian Information Criterion (BIC). All statistical analyses were performed using R version 4.6.1 (R Foundation for Statistical Computing, Vienna, Austria), with negative binomial models fitted using the glm.nb function from the MASS package.

## Results

### Intercontinental introduction and establishment of local mpox transmissions in South Korea

The global spread of mpox was driven by human-to-human transmission, characterized by APOBEC3-induced mutations [5] and intercontinental dissemination. Identified as MPXV clade IIb, sub-lineage B.1.3, the virus was the central strain in the outbreak centred in Europe in August 2022. It circulated in Europe and the United States before being identified in Asia, where it underwent a bottleneck effect and potential evolutionary modification under selective environmental pressure. Genetically clustered MPXVs were designated as the C.1 sub-lineage, which subsequently continued to circulate in Europe and other regions (appendix p 2). In August 2022, mpox outbreaks peaked in the United States and Europe, whereas incidence rates were much lower in Japan and South Korea. By early 2023, Japan experienced a surge in cases linked to MPXV strains from the United States and Europe, and in May 2023, South Korea experienced an outbreak associated with Japan. By September 2023, the incidence rates in Asia had declined, whereas those in Europe and the United States increased slightly by early 2024 and have since remained steady. In South Korea, four mpox cases were reported after the first patient was identified in June 2022, following importation from Europe. Excluding the case related to medical infection, all others were associated with overseas importation through sexual contact. However, in March 2023, new cases began to emerge, with an endemic peak in May. Mpox cases in South Korea increased from 4 in 2022–151 in 2023, even as the WHO declared the end of the PHEIC amid a global decline (Figure 1A). The median age of the patients from June 2022 to December 2023 was 32 years (interquartile range [IQR], 8·75) (Figure 1D). Most of these individuals were males (152 of 155, 98·1%) and were infected through sexual contact. Among patients reported in 2023, 17 were born in South Korea before 1978, indicating they had likely received the smallpox vaccine. The remaining patients belonged to a generation less likely to have been vaccinated against smallpox. Since 2022, the incubation period of mpox cases in South Korea has been 11·6 d on average (95% confidence interval, 10·376–12·756), exceeding the 9·8 d reported in the United Kingdom in 2022 [13] and comparable to the 12·5 d reported in Italy (Figure 1E) [14]. Most patients exhibit typical mpox symptoms, including skin lesions, although rare cases present with atypical symptoms such as ocular disorders. Notably, the occurrence of mpox in individuals living with human immunodeficiency virus (HIV) is associated with more severe symptoms, particularly among those with compromised immune function. The 2023 rise in locally acquired mpox cases – mainly among individuals without overseas travel history – suggested regionally uneven transmission, likely explained by social connectivity and exposure patterns (Figure 1B, C). The MPXV that fuelled the 2022 global outbreak spread rapidly worldwide, leveraging the epidemiological characteristics of heavy-tailed sexual contact networks; a small subset of individuals with disproportionately many partners were pivotal in sustaining transmission, particularly among the MSM community [15]. Within this framework, the mpox outbreak in South Korea may have been driven by similar epidemiological dynamics. Genome sequences from 142 PCR-positive individuals (appendix p 3) enabled the identification of circulating MPXV strains and provided insights into their transmission dynamics and mutational patterns. In 2022, mpox cases associated with travel to Europe were confirmed as clade IIb sub-lineage B.1.1 MPXV infections, which were prevalent in Europe at that time. Positive cases from the same year, including a healthcare worker, were identified as imported infections caused by the H.1 lineage [16]. Genomic analysis of 134 samples collected in 2023–2024 revealed that most cases in South Korea belonged to the C.1 lineage; the remaining were C.1.3 (n = 6), E4 (n = 2), and C.1.1 (n = 1). Subsequently, in 2025, E.4 predominated (3 of 4 cases) (Figure 2A). The asymmetry in lineage distribution had several important implications. The perpetuation of anonymity and intimate interactions emerged as pivotal factors precipitating the extensive propagation of a specific viral lineage. Approximately 70% of the cases analyzed through genomic analysis were linked to close contact with anonymous individuals, facilitated through platforms such as social networking services or visits to specific spaces, leading to increased informal encounters and transmission in a highly networked manner (Figure 3A, C).

**Figure 1.**
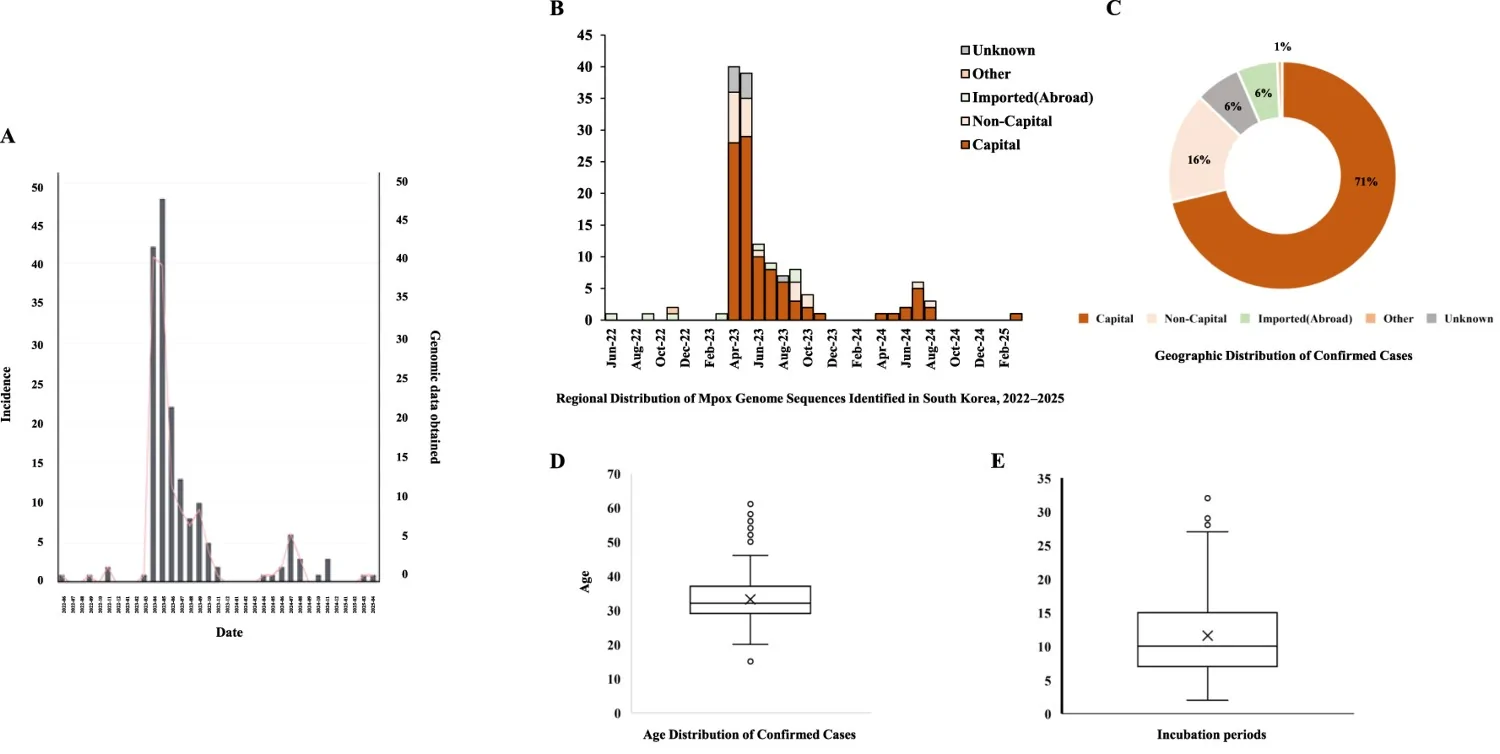
Epidemiological characteristics of the mpox outbreak in South Korea between 2022–25. (A) Monthly number of confirmed mpox cases (bars) and number of sequenced viral genomes (line). (B) Regional distribution of sequenced mpox genomes. (C) Geographical distribution of confirmed cases by administrative regions. (D) Age distribution of confirmed cases. (E) Estimated incubation periods among confirmed cases.

**Figure 2.**
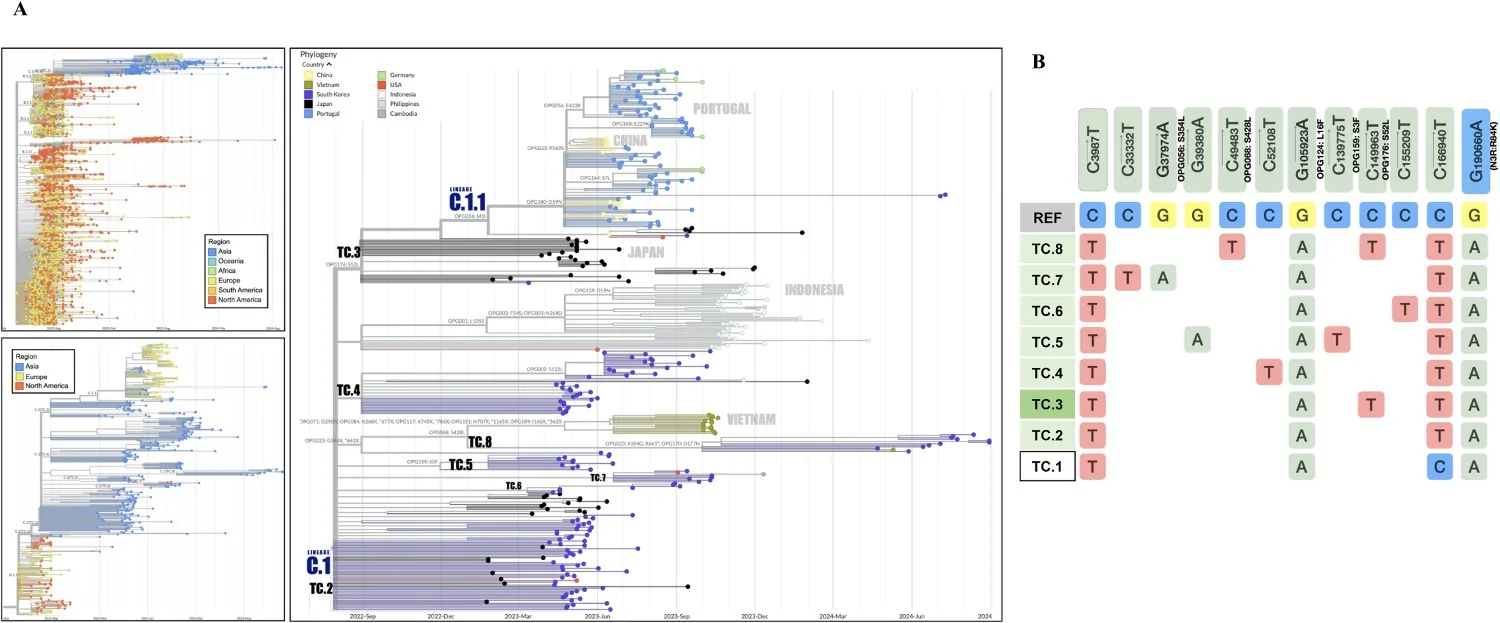
Phylogenetic analysis and clustering of MPXV genomes from the 2022–2025 Mpox outbreak in South Korea, as of October 1, 2025. (A) Time-resolved phylogenetic tree of 142 mpox virus genomes from South Korea, constructed using the Nextstrain CLI (v7.2.0). Sequences were aligned with CGW20 against the reference genome NC_063383. A single dominant clade associated with local transmission is highlighted, showing limited diversification. (B) Shared nucleotide variants identified using Snippy and visualized with SNIPIT, relative to the reference genome NC_063383.

**Figure 3.**
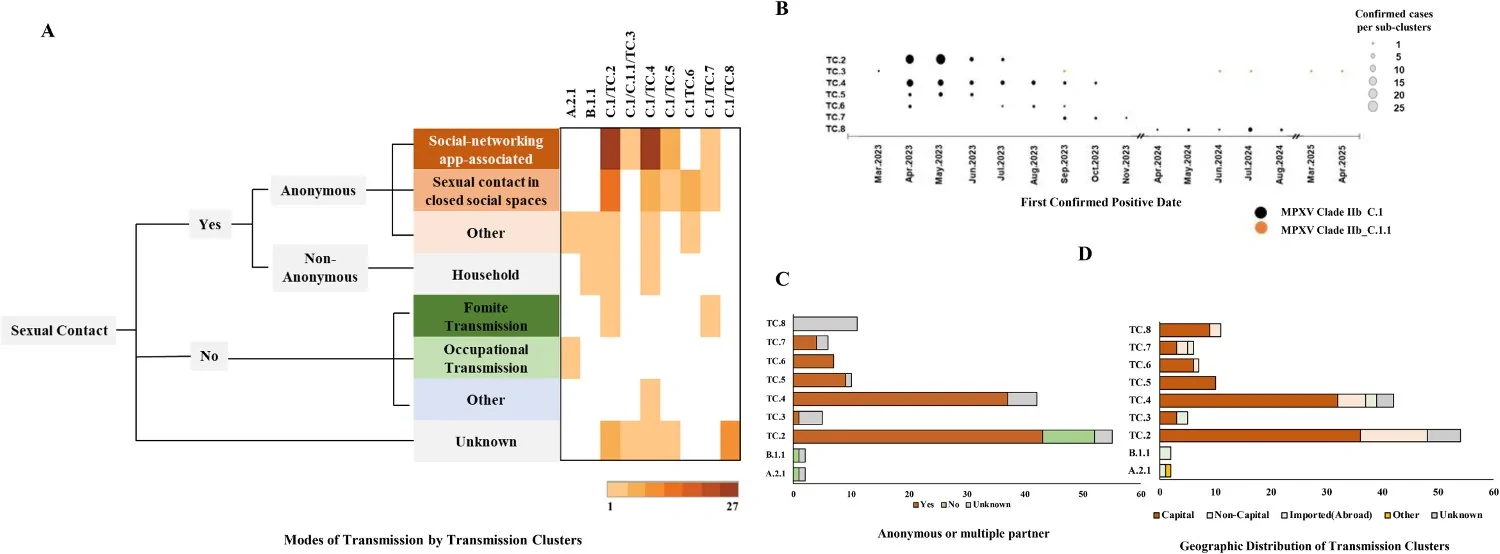
Transmission dynamics and clustering characteristics of the mpox outbreak in South Korea, 2022–25. Genomic clusters were defined primarily based on phylogenetic topology, with cluster-specific shared mutation patterns also considered, as described in the Methods. Epidemiological characteristics, including anonymous contacts, multiple sexual partners, and app-mediated interactions, were subsequently evaluated across the identified genomic clusters. (A) Heatmap illustrating the dominant transmission modes associated with each genomic cluster. (B) Temporal landscape of mpox virus sub-clusters in South Korea between 2023 and 2025, inferred from genomic and epidemiological data. (C) Proportion of individuals within each cluster reporting anonymous contacts and/or multiple sexual partners. (D) Geographical distribution of major transmission clusters, illustrating their spatial distribution across South Korea.

### Genomic clustering reveals transmission dynamics and mutation emergence during sustained mpox spread

To investigate genomic relationships among circulating MPXV strains during sustained transmission in South Korea, phylogenetic analysis identified eight genomic clusters, including one outgroup and seven domestics genomic subclusters. Genomic subclusters were defined based on phylogenetic topology, branching structure, and characteristic branch-defining mutations identified within the Nextstrain framework. Monophyletic groups generally comprising five or more cases were designated as genomic subclusters, although phylogenetically distinct groups comprising fewer than five cases were also retained as independent subclusters. The identified branch-defining mutations were subsequently validated using Snippy and visualized with SNIPIT (Figure 2A–B). Each domestic subcluster comprised 5–53 individuals, forming distinct clusters (Figure 2A). All MPXV genomes identified in South Korea from 2023 to 2025, including that of the index case, harboured the G190660A (N3R:R84 K) mutation – originally found in the B.1.3 lineage – indicating a shared common ancestor with the MPXV strains responsible for the global outbreak, including that in Europe (Figure 2B). Notably, genomic surveillance of MPXV isolates from South Korea identified the G105923A (OPG124:L16F) mutation in all confirmed cases, including the index patient. The presence of this shared mutation suggests a common genomic origin among the Korean cases and is consistent with a limited number of closely related viral introduction events followed by subsequent domestic transmission. The consistent detection of this mutation across cases also indicates localized and rapid transmission in high-contact settings following its initial entry into the population. At the time of the index case, G105923A was absent in the MPXV genomes from Germany, the USA, and Canada but was identified in those from the TC.1, as defined in this study, which includes Japan, Taiwan, and Belgium (Figure 2B). Moreover, a phylogenetic analysis revealed a close genetic relationship between the MPXV strains from South Korea and Japan. These findings enabled the estimation of the features of introduction and transmission at local transmission onset. When estimating infection dates, TC.3 was identified as the earliest. This subcluster comprises strains linked to Japan and China, characterized by a shortened incubation period and rapid diagnostic confirmation, with transmission restricted to a small number of individuals (n = 2, March–September 2023) (Figures 2 and 3B). This observation suggests that early response measures, such as quarantining individuals with a history of overseas travel, may have effectively mitigated further viral transmission within this group. In South Korea, the TC.2 subcluster (n = 53, April–July 2023) spread and became predominant. The initial introduction of TC.2 likely occurred within a community characterized by specific sexual networks, where anonymity – often mediated through app-based platforms – facilitated rapid transmission. This epidemiological context coincided with the substantial expansion of TC.2, which formed the largest genomic cluster during the South Korea’s mpox outbreak (Figures 2 and 3). Indeed, 79% (67 of 85 cases) of contacts in the TC.2 subcluster involved anonymous partners, further supporting this transmission pattern (Figure 3C). This group includes the common B.1.3 mutation G190660A (OPG050:R26Q and OPG056:S354L), along with the C3987 T, G105923A (OPG124:L16F), and C166940 T mutations, collectively accounting for over 38% of the confirmed cases in 2023 (Figure 2B). The TC.4 subcluster (n = 42, April–October 2023), which diverged from the TC.2 subcluster, similarly exhibited characteristics associated with social networks and anonymity, having circulated for a prolonged period in South Korea (Figures 2 and 3B). Among contacts linked to TC.4, 86% (66 of 77 cases) involved interactions with anonymous partners, many of which were single encounters. These factors posed challenges for accurately tracing infection pathways and complicated public health response efforts. TC.4 acquired the C52108 T non-synonymous mutation, along with the mutations shared with TC.2 (Figures 2 and 3). The TC.6 subcluster (n = 7, April–September 2023), phylogenetically diverging from TC2, was associated with transmission occurring in social spaces frequented by individuals within a specific sexual network. These interactions were predominantly characterized by anonymity, contributing to the formation of a distinct transmission cluster, with all associated contacts reported as anonymous (100%, 17 of 17 cases) (Figures 2 and 3). Most of these (6 of 7, 85·7%) involved individuals living with HIV who harboured the C155209 T non-synonymous mutation, a common variant also found in the TC.2 subcluster. This mutation emerged concurrently with the early TC.2 subcluster. Some cases were epidemiologically linked to Taiwan and Thailand (Figure 2A). Similar to other subclusters, the TC.5 subcluster (n = 10, April–June 2023) was predominantly driven by sexual contact with anonymous partners, mostly facilitated through social networking platforms (Figure 3). The high proportion of anonymous contacts complicated the assessment of transmission dynamics, and index cases had interacted with partners of various nationalities. The TC.5 subcluster contains both synonymous and non-synonymous mutations, bearing implications for viral protein function and pathogenicity. The TC.5 subcluster includes mutations found in TC.2, including G39380A and C139775 T (OPG159:S3F) (Figure 2B). C139775 T is located within the OPG159 genomic region, which encodes the A33R protein – a key factor in efficient cell-to-cell spread and the formation of actin-containing microvilli [17,18]. The TC.5 subcluster included a case of tecovirimat resistance identified in a patient with uncontrolled HIV [19], who carried a non-synonymous mutation within the genomic region encoding A33R. Furthermore, an atypical clinical presentation, namely an ocular disorder, was documented in this group. The first case of TC.7 (n = 6, September–November 2023), which emerged toward the end of 2023, involved travel history abroad. TC.7 accumulated genetic mutations through local transmission via human-to-human contact in South Korea (Figure 3). These include mutations found in TC.2, which is considered the main cause of cases in South Korea and bears the C33332 T and G37974A mutations. G37974A is a non-synonymous mutation located in the OPG050 (R26Q) and OPG056 (S354L) genomic regions (Figure 2B). The S354L mutation is associated with the intracellular transport of enveloped viruses to the cell surface through microtubule transport [20]. No confirmed mpox cases were reported in South Korea from the end of 2023 to April 2024. However, owing to ongoing local silent transmission, there may have been undetected cases leading to transmission. South Korea remained without confirmed cases until the first patient was reported in April 2024. By September 2024, genomes were obtained from 13 positive cases. Of the 13 cases, 11 were classified in the TC.8 subcluster of the C.1 lineage. Genetic analyses confirmed that the newly identified subcluster TC.8, which branched from the TC.2 subcluster, led to the 2023 mpox outbreak in South Korea. The TC.8 subcluster harbours common variants of TC.2 along with additional non-synonymous mutations in OPG068, encoding an IMV membrane protein, and OPG176, a TLR4 pathway inhibitor involved in host immune modulation (Figure 2B). The remaining two cases belonged to the C.1.1 lineage; when clustered by common mutations, they clustered with TC.3, which was associated with Japan and China and marked the beginning of the mpox outbreak in South Korea. These two cases exhibited a considerable increase in SNPs, along with several mutations differing from previously reported findings, similar to the typical C.1.1 lineage [21]. Notably, the first confirmed mpox case of 2025, reported in March, was characterized by further genomic diversification and a distinct mutational profile diverging from the dominant domestic strain (Figure 4A, B). Excluding the outgroup, the distribution of the seven genomic clusters exhibited substantial overdispersion, with both the variance-to-mean ratio (VMR) and Pearson dispersion statistic estimated at 21.29. The negative binomial model provided a markedly better fit than the Poisson model (AIC, 59.75 vs. 144.34; BIC, 59.64 vs. 144.28). The estimated negative binomial dispersion parameter was θ = 1.429 (SE = 0.747). Together, these findings indicate marked heterogeneity in the sizes of the observed genomic clusters.

**Figure 4.**
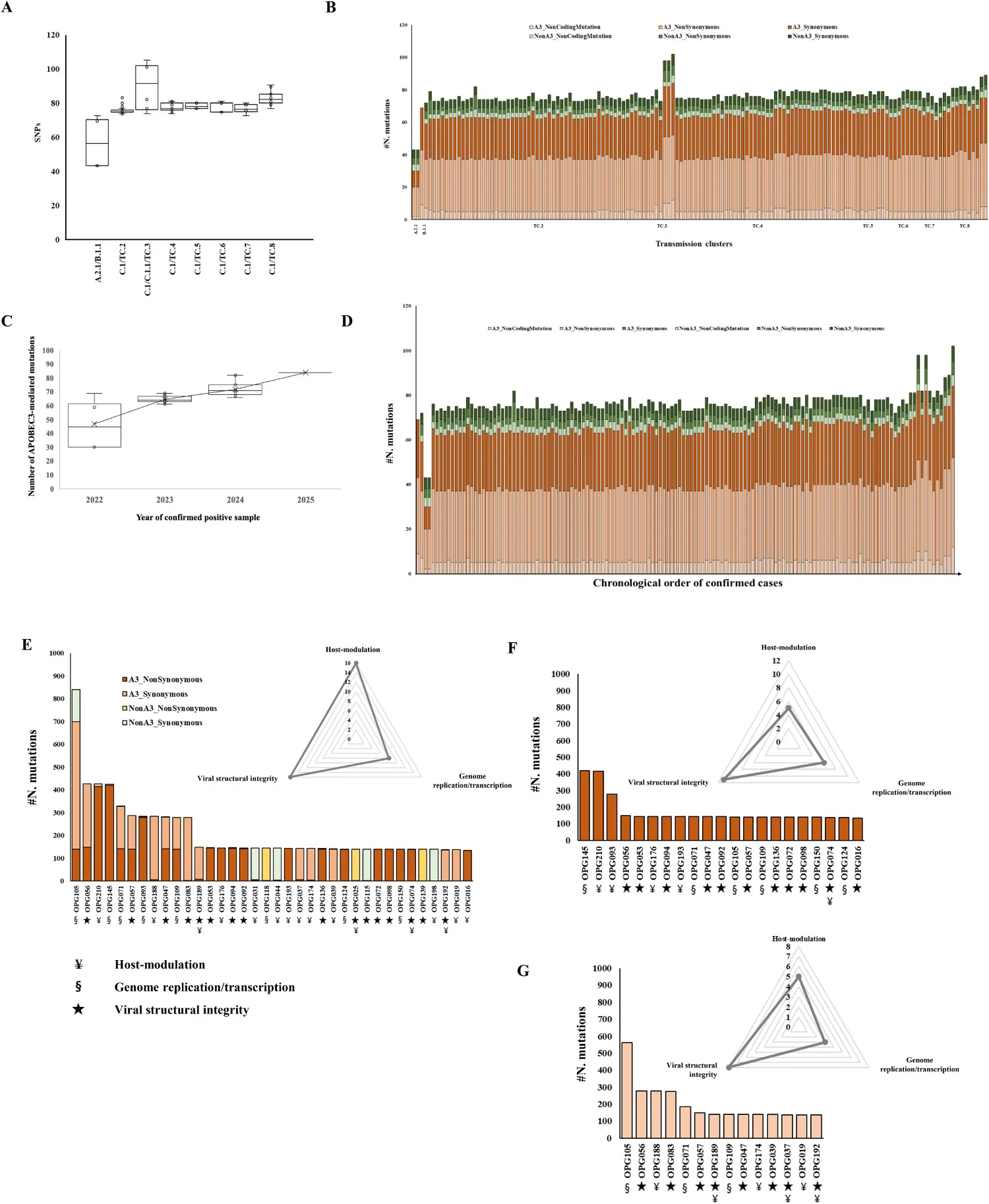
APOBEC3-associated mutational landscape and genomic diversification of mpox virus in South Korea, 2022–2025. These findings indicate that APOBEC3 activity substantially shapes the observed genomic diversity during the outbreak, with mutation accumulation concentrated in functionally distinct genomic regions. (A) Genomic diversity (single nucleotide polymorphisms; SNPs) among genomic clusters, illustrating intra-cluster variation. (B) Classification of mutations by type, including APOBEC3-associated mutations and non-APOBEC3-related nonsynonymous substitutions. (C, D) Temporal trends in APOBEC3-associated mutations from 2022 to 2025, showing a steady increase in prevalence over time. (E–G) Identification of mutation hotspots based on aggregated mutation profiles from 140 mpox genomes. Hotspots were defined as genomic regions with >100 mutations, excluding repetitive or ambiguous regions such as ITRs. Functional annotation of these regions was performed using ViralZone categories.

### Genomic hotspots of APOBEC3-associated mutations and nonsynonymous divergence during sustained transmission

As observed in outbreaks across multiple countries, accumulation of genomic variants during an outbreak in South Korea was strongly associated with APOBEC3 deaminase activity. Genomic surveillance revealed a marked increase in APOBEC3-mediated mutations from 2022 to 2025 (Figure 4C, D). Mutation profiling of all MPXV genomes confirmed in South Korea during this period revealed considerable heterogeneity in pairwise dN/dS estimates across distinct genomic regions (Table 1). Among genes containing identified mutations, several exhibited elevated nonsynonymous relative to synonymous divergence. Notably, genes associated with viral structural integrity, such as *OPG056* (dN/dS  =  2·7) and *OPG153* (dN/dS  =  4.5 [95% CI: 1·3–6·0]), showed relatively high pairwise dN/dS values. Given the established role of APOBEC3-mediated editing in introducing such mutations, these findings suggest that APOBEC3 activity may contribute to genomic diversification by generating genetic variation during sustained human-to-human transmission. Based on this surveillance, hotspots of APOBEC3-mediated mutations were mainly identified in genomic regions associated with host modulation, genome replication/transcription, and virion structural integrity, including genes involved in virion assembly, stability, and egress (Table 1). These patterns indicate that APOBEC3-associated mutations were distributed across multiple functional categories during sustained human-to-human transmission. Notably, mutations induced by APOBEC3 were most frequently observed in the ankyrin repeat protein region, which is critical for viral structural integrity (Figure 4E, F). These mutations are a double-edged sword for the virus. Although they contribute to immune evasion, they also pose a risk to the virus’s structural integrity, potentially compromising its infectivity and transmission efficiency. Indeed, mutations in the ankyrin repeat protein region are associated with reduced viral virulence [22], supporting the notion that structural compromise can attenuate the virus. However, certain mutations may facilitate viral adaptation to new hosts by enabling efficient transmission under diverse environmental conditions. Specifically, the S354L non-synonymous mutation in the OPG056 region observed in TC.7. OPG056 has been associated with microtubule-mediated of the intracellular enveloped virions to the cell surface, a process involved in viral egress and cell-to-cell spread [23,24]. However, the functional consequence of the S354L substitution itself was not evaluated in this study. APOBEC3-associated mutations were also frequently observed in genomic regions associated with host immunity, further indicating that the virus evolved continuously to evade host immunity through repeated human-to-human transmission.

**Table 1.** Genes exhibiting elevated nonsynonymous divergence(dN/dS ≥ 1) in MPXV during the 2022–2025 outbreak in South Korea.

OrthoPox Genes	Vaccinia Copenhagen homolog	Category	dN/dS [A3-related mutation rate]	Product
OPG153	**A26L**	**Viral structural integrity (Surface protein)**	**1.3∼6(4.5)** [50%]	**A26L/A30L membrane association protein**
OPG145	A18R	Genome replication/transcription	1.7 [100%]	Transcription factor
OPG125	D13L	Viral structural integrity (Surface protein)	1.3	Intracellular mature virion coat
OPG071		Genome replication/transcription	1.2 [99%]	DNA polymerase
OPG056	**F12L**	**Viral structural integrity (Assembly, budding)**	2.7 [100%]	**Actin tail budding**
OPG040	K2L	Host-modulation	2 [100%]	Superinfection exclusion
OPG023		Host-modulation	1.2 [100%]	Ankyrin repeat protein
OPG002	C22L	Host-modulation	1.02/1.09 [100%]	Chemokine binding

Genes with dN/dS values ≥1 were identified based on pairwise dN/dS estimates calculated with KaKs Calculator (YS method), with the Clade I genome (KJ642616) as the reference. This reference was selected to provide sufficient sequence divergence for exploratory assessment of elevated nonsynonymous divergence. The proportion of mutations attributed to APOBEC3-mediated editing is indicated in parentheses next to each dN/dS value.

## Discussion

This study provides a nationwide genomic perspective on how the mpox virus spread and evolved in South Korea, a non-endemic setting where sustained human-to-human transmission occurred after intercontinental introduction. The 2022–2025 mpox outbreak marked a shift from geographically restricted zoonotic spillover toward prolonged human transmission across international networks. South Korea recapitulated this global transition at a regional scale, providing an opportunity to examine how structured transmission networks influence viral diversification and potential adaptation in human populations. Our findings show that the Korean outbreak was not characterized by widespread random dissemination, but rather by sustained transmission within structured and partially epidemiologically obscured contact networks. The identification of a shared mutation, L16F, across domestic cases supports a common transmission origin, followed by continued local spread. This pattern is consistent with previous observations that mpox transmission can be strongly shaped by defined social and contact networks, particularly when transmission occurs through contacts that are difficult to fully capture through conventional epidemiological investigation [25]. In this context, the Korean outbreak represents a setting in which transmission was not only geographically localized but also socially structured and partially hidden from traditional surveillance systems. A major feature of the Korean outbreak was the uneven expansion of domestic transmission clusters. Although multiple genomic clusters were identified, dominant cluster accounted for a substantial proportion of domestic cases, whereas most other clusters remained relatively limited in size. This pattern suggests that local transmission was asymmetric rather than evenly distributed across the identified genomic clusters. While formal inference of individual-level heavy-tailed transmission was beyond the scope of this study, the disproportionate expansion of a dominant cluster is compatible with transmission heterogeneity within contact networks, in which a limited number of transmission chains may contribute disproportionately to onward spread. In South Korea, the observed epidemiological characteristics, including social anonymity and epidemiologically unrecognized contacts, may have contributed to such transmission heterogeneity and facilitated undetected transmission within localized subpopulations. The combination of interconnected contact patterns, partial anonymity, and sustained human-to-human spread provides an important framework for understanding the mpox virus evolution in this non-endemic setting. In endemic regions, repeated zoonotic spillover events and relatively short human transmission chains may obscure or limit the observation of prolonged human-adapted evolutionary trajectories. By contrast, sustained local circulation in South Korea created an opportunity to observe viral microevolution during continued transmission in a human population. In this context, genomic clustering was useful not only for characterizing transmission patterns but also for tracing the emergence and spread of cluster-specific mutations during the outbreak. A key finding of this study was the progressive accumulation of APOBEC3-associated mutations over the course of the outbreak. These mutations likely contributed to viral diversification during sustained human-to-human transmission. Importantly, recurrent genomic hotspots of APOBEC3-associated mutations and elevated nonsynonymous divergence were not randomly distributed across the genome but were frequently observed in genes associated with viral structure and morphogenesis, replication, and host modulation. Although these functional categories are biologically distinct, they encompass processes involved in viral replication and interactions with the host environment. Alterations in genes involved in surface protein composition, virion assembly, and egress may influence antigenic exposure, particle stability, and intercellular spread; whereas variation in replication-associated genes may reflect continued genomic diversification during sustained transmission Changes in host-modulatory genes may also influence viral interaction with host immune responses. Together, these findings suggest that genomic changes affecting virus-host interactions may constitute an important component of MPXV diversification during sustained human transmission. However, APOBEC3-mediated mutation should not, by itself, be interpreted as direct evidence of adaptation. Rather, APOBEC3 editing may contribute to genetic diversity that can subsequently be subject to selection, with some variants potentially being retained during continued transmission. The overlap between APOBEC3-associated hotspots, regions showing elevated nonsynonymous divergence, and genes involved in immune interaction, viral morphogenesis, or host modulation is therefore consistent with, but does not demonstrate, evolutionary changes associated with sustained human transmission. Functional validation will be required to determine whether these mutations alter viral fitness, immune evasion, virion stability, cell-to-cell spread, or transmission efficiency. Our findings also have important public health implications. Transmission within interconnected and partially anonymous contact patterns may complicate containment efforts and provide opportunities for continued viral circulation and genomic diversification. This is particularly relevant for pathogens such as the mpox virus, which were historically maintained through zoonotic transmission but are now capable of sustained human-to-human spread in non-endemic regions. In such settings, conventional epidemiological surveillance may fail to identify cryptic transmission chains, especially when social anonymity limits contact tracing. Genomic surveillance can therefore provide complementary information by identifying genetically related cases and dominant genomic clusters and by monitoring mutation patterns that may have biological or public health relevance.

This study has several limitations. First, functional validation of the identified mutations was not performed; therefore, their effects on viral phenotype, immune evasion, or transmission fitness remain uncertain. Second, sampling bias may have influenced cluster detection and the apparent distribution of mutations across transmission groups. Third, although the uneven expansion of domestic clusters suggests asymmetric transmission, this study was not designed to directly estimate individual-level transmission heterogeneity. Accordingly, the dispersion parameter estimated from cluster-size modelling should be interpreted as a measure of cluster-level expansion heterogeneity rather than as direct evidence of individual-level superspreading. Fourth, pairwise dN/dS estimates in this study should be interpreted as exploratory evolutionary metrics rather than formal tests of positive selection. Because the analysis was restricted to genes containing identified mutations, relied on pairwise comparisons with a phylogenetically divergent reference genome, and did not incorporate phylogenetic codon-based models, the resulting estimates should not be considered definitive evidence of adaptive evolution. Nevertheless, these exploratory estimates may help identify genes warranting further investigation using more comprehensive evolutionary analyses and functional studies. Future studies incorporating functional assays, more detailed epidemiological data, and broader international genomic comparisons will be important for defining the biological significance of APOBEC3-associated mutations and their contribution to mpox virus adaptation.

In conclusion, our study demonstrates that sustained mpox transmission in South Korea provided an opportunity to characterize genomic diversification during continued human-to-human transmission. The observed genomic clustering and progressive accumulation of APOBEC3-associated mutations illustrate the dynamic genomic changes occurring during prolonged viral circulation. These findings underscore the value of integrating genomic and epidemiological surveillance systems to characterize transmission patterns and monitor viral evolution, particularly in non-endemic settings experiencing sustained human-to-human transmission.

## Data sharing statement

The mpox virus genomes newly generated in this study, as of September 2025, are publicly available in GenBank. The accession numbers for the individual sequences are provided in Appendix p 3. All shared data is accessible without any restrictions.

## Supplementary Material

supplementary_material.docx
