# Supplementary material for: Unravelling the transmission dynamics and evolutionary trajectory of mpox in South Korea: 2022–2025 outbreak
Source: Emerg Microbes Infect. 2026 Oct 7;15(1):2746255. doi: 10.1080/22221751.2026.2746255 (PMC13686212; doi:10.1080/22221751.2026.2746255)
Supplement: Supplementary file 1 [file TEMI_A_2746255_SM8977.docx]

**Supplementary material**

**Supplementary table 1. Functional classification of mpox virus genes based on the NC_063383 reference genome.**

| OrthoPox genes | Vaccinia Copenhagen homolog | Functions | Category |
| --- | --- | --- | --- |
| OPG001 | C23L | Chemokine binding | Host-modulation |
| OPG002 | C22L | TNF receptor | Host-modulation |
| OPG003 | C19L | Ankyrin | Host-modulation |
| OPG015 |  | Ankyrin | Host-modulation |
| OPG019 | C11R | Secreted EGF-like | Host-modulation |
| OPG021 |  | Zinc finger | Host-modulation |
| OPG022 |  | IL-18 binding protein | Host-modulation |
| OPG023 |  | Ankyrin | Host-modulation |
| OPG025 | C9L | Ankyrin | Host-modulation |
| OPG027 | C7L | IFN inhibitor | Host-modulation |
| OPG029 | C6L |  | Host-modulation |
| OPG031 | D13L | IL-1R agonist | Host-modulation |
| OPG034 | C1L |  | Host-modulation |
| OPG035 | N1L |  | Host-modulation |
| OPG036 | N2L | TLR inhibitor | Host-modulation |
| OPG037 | M1L | Apoptosis inhibitor | Host-modulation |
| OPG038 | M2L | NFkB inhibitor | Host-modulation |
| OPG039 | K1L | NFkB inhibitor | Host-modulation |
| OPG040 | K2L | Superinfection exclusion | Host-modulation |
| OPG042 | K4L |  | Unknown |
| OPG043 | K5L |  | Unknown |
| OPG044 | K7R |  | Unknown |
| OPG045 | F1L | Caspase-9 inhibitor | Host-modulation |
| OPG046 | F2L |  | Unknown |
| OPG047 | F3L |  | Unknown |
| OPG048 | F4L | Acid Nuclease enzyme | Genome replication/transcription |
| OPG049 | F5L |  | Surface protein |
| OPG050 | F6L |  | Unknown |
| OPG051 | F7L |  | Unknown |
| OPG052 | F8L |  | Unknown |
| OPG053 | F9L |  | Surface protein |
| OPG054 | F10L | Kinase enzyme | Unknown |
| OPG055 | F11L | Budding | Assembly, budding |
| OPG056 | F12L | Actin tail budding | Assembly, budding |
| OPG057 | F13L |  | Surface protein |
| OPG058 | F14L |  | Unknown |
|  |  |  |  |
| OPG059 | F14.5L |  | Unknown |
| OPG060 | F15L |  | Unknown |
| OPG061 | F16L |  | Unknown |
| OPG062 | F17R |  | Unknown |
| OPG063 | E1L | Poly A polymerase | Genome replication/transcription |
| OPG064 | E2L | IEV morphogenesis | Assembly, budding |
| OPG065 | E3L | IFN resistance | Host-modulation |
| OPG066 | E4L | RNA polymerase subunit | Genome replication/transcription |
| OPG068 | E6R |  | Assembly, budding |
| OPG069 | E7R |  | Unknown |
| OPG070 | E8R |  | Assembly, budding |
| OPG071 |  | DNA polymerase | Genome replication/transcription |
| OPG072 | E10R | Sulfhydryl oxidase | Surface protein |
| OPG073 | E11L |  | Assembly, budding |
| OPG074 | O1L | Membrane protein | Structural integrity, Host-modulation |
| OPG075 | O2L |  | Assembly, budding |
| OPG077 | I1 | DNA binding | Genome replication/transcription |
| OPG078 | I2L |  | Surface protein |
| OPG079 | I3L | ssDNA binding | Genome replication/transcription |
| OPG080 | I4L | Ribonuclease reduction enzyme | Genome replication/transcription |
| OPG081 | I2L |  | Surface protein |
| OPG082 | I6L | Telomere binding | Genome replication/transcription |
| OPG083 |  | Virion core | Assembly, budding |
| OPG084 | I8R |  | Genome replication/transcription |
| OPG085 | G1L | Protease | Assembly, budding |
| OPG086 | G3L |  | Surface protein |
| OPG087 | G2R | Trans-acting factor | Genome replication/transcription |
| OPG088 | G4L | Sulfhydryl oxidase | Assembly, budding |
| OPG089 | G5R |  | Genome replication/transcription |
| OPG090 | G5.5R | RNA polymerase subunit | Genome replication/transcription |
| OPG091 | G6R |  | Unknown |
| OPG092 | G7L |  | Assembly, budding |
| OPG093 | G8R | Trans-acting factor | Genome replication/transcription |
| OPG094 | G9R |  | Surface protein |
| OPG095 | L1R |  | Surface protein |
| OPG096 | L2R | Viral membrane-associated protein | Assembly, budding |
| OPG097 | L3L |  | Assembly, budding |
| OPG098 | L4R | Virion core | Assembly, budding |
| OPG099 | L5R |  | Surface protein |
| OPG100 | J1R |  | Surface protein |
| OPG101 | J2R | Thymidine kinase | Genome replication/transcription |
| OPG102 | J3R | 2'O Methyl transferase | Genome replication/transcription |
| OPG103 | J4R | DNA-dependent RNA polymerase | Genome replication/transcription |
| OPG104 | J5L |  | Unknown |
| OPG105 | J6R | RNA polymerase subunit | Genome replication/transcription |
| OPG106 | H1L | Interferon inhibition | Host-modulation |
| OPG107 | H2R |  | Surface protein |
| OPG108 | H3L |  | Surface protein |
| OPG109 | H4L | RNA polymerase subunit | Genome replication/transcription |
| OPG110 | H5R | Viral Late Transcription Factor 4 | Genome replication/transcription |
| OPG111 | H6R | DNA Topoisomerase | Genome replication/transcription |
| OPG112 | H7R | Viral membrane-associated protein | Genome replication/transcription |
| OPG113 | D1R | mRNA-capping enzyme large subunit | Genome replication/transcription |
| OPG114 | D2L | Virion core | Assembly, budding |
| OPG115 | D3R | Virion core | Assembly, budding |
| OPG116 | D4R | DNA polymerase family | Genome replication/transcription |
| OPG117 | D5R | DNA primase | Genome replication/transcription |
| OPG118 | D6R | Viral Early Transcription Factor Small Subunit | Genome replication/transcription |
| OPG119 | D7R | RNA polymerase subunit | Genome replication/transcription |
| OPG120 | D8L |  | Surface protein |
| OPG121 | D9R | mRNA decapping | Genome replication/transcription |
| OPG122 | D10R | mRNA decapping | Genome replication/transcription |
| OPG123 | D11L | DNA-dependent ATPase | Genome replication/transcription |
| OPG124 | D12L | mRNA-capping | Genome replication/transcription |
| OPG125 | D13L | Intracellular mature virion coat | Surface protein |
| OPG126 | A1L | Vaccinia Late Transcription Factor 2 | Genome replication/transcription |
| OPG127 | A2L | Late Transcription Factor 3 | Genome replication/transcription |
| OPG128 | A2.5L |  | Genome replication/transcription |
| OPG129 | A3L | Virion core | Assembly, budding |
| OGP130 | A4L | Virion core | Assembly, budding |
| OPG131 | A5R | DNA polymerase subunit | Genome replication/transcription |
| OPG132 | A6L | Viral membrane-associated protein | Assembly, budding |
| OPG133 | A7L | Vaccinia Early Transcription Factor Large subunit | Genome replication/transcription |
| OPG134 | A8R | Vaccinia Intermediate Transcription Factor 3 | Genome replication/transcription |
| OPG135 | A9L |  | Surface protein |
| OPG136 | A10L | Major core | Assembly, budding |
| OPG137 | A11R | Viral membrane-associated protein | Assembly, budding |
| OPG138 | A12L | Core cleavage | Assembly, budding |
| OPG139 | A13L |  | Surface protein |
| OPG140 | A14L |  | Surface protein |
| OPG141 | A15.5L |  | Surface protein |
| OPG142 | A15L |  | Surface protein |
| OPG143 | A16L |  | Surface protein |
| OPG144 | A17L |  | Surface protein |
| OPG145 | A18R | Transcription factor | Genome replication/transcription |
| OPG146 | A19L |  | Surface protein |
| OPG147 | A21L |  | Surface protein |
| OPG148 | A20R | DNA polymerase processivity factor | Genome replication/transcription |
| OPG149 | A22R |  | Genome replication/transcription |
| OPG150 | A23R | Vaccinia Intermediate Transcription Factor 3 | Genome replication/transcription |
| OPG151 | A24R | RNA polymerase subunit | Genome replication/transcription |
| OPG153 | A26L |  | Surface protein |
| OPG154 | A27L |  | Surface protein |
| OPG155 | A28L |  | Surface protein |
| OPG156 | A29L | RNA polymerase subunit | Genome replication/transcription |
| OPG157 | A30L |  | Surface protein |
| OPG158 | A30.5L |  | Unknown |
| OPG159 | A31R |  | Unknown |
| OPG160 | A32L | DNA packaging | Assembly, budding |
| OPG161 | A33R |  | Surface protein |
| OPG162 | A34R |  | Surface protein |
| OPG163 | A35R | MHC inhibitor | Host-modulation |
| OPG164 | A36R |  | Surface protein |
| OPG165 | A37R |  | Unknown |
| OPG166 |  |  | Unknown |
| OPG167 | A38L |  | Surface protein |
| OPG170 | A41L | Chemokine binding | Host-modulation |
| OPG171 | A42R |  | Unknown |
| OPG172 | A43R |  | Host-modulation |
| OPG173 | A43.5R |  | Unknown |
| OPG174 | A44L | Steroid metabolism | Host-modulation |
| OPG175 | A45R | Virion core | Assembly, budding |
| OPG176 | A46R | Interleukin inhibitor | Host-modulation |
| OPG178 | A48R | Thymidine kinase | Genome replication/transcription |
| OPG180 | A50R | DNA ligase | Genome replication/transcription |
| OPG181 | A51R |  | Unknown |
| OPG185 | A56R |  | Surface protein |
| OPG187 | B1R | Serine/Threonine Kinase | Assembly, budding |
| OPG188 | B2R | Poxin | Host-modulation |
| OPG189 | B4R | Ankyrin | Host-modulation |
| OPG190 | B5R |  | Surface protein |
| OPG191 | B6R |  | Host-modulation |
| OPG192 | B7R | Ankyrin | Host-modulation |
| OPG193 | B8R | Interferon-gamma binding | Host-modulation |
| OPG195 |  |  | Host-modulation |
| OPG197 | B11R |  | Host-modulation |
| OPG198 | B12R |  | Host-modulation |
| OPG199 | K2L | Interleukin inhibitor | Host-modulation |
| OPG200 | B15R | TLR inhibitor | Host-modulation |
| OPG204 | B19R | IFN receptor | Host-modulation |
| OPG205 | B20R | Ankyrin | Host-modulation |
| OPG208 | K2L | Apoptosis inhibitor | Host-modulation |
| OPG209 | C14L |  | Host-modulation |
| OPG210 |  | T cell suppressor | Host-modulation |
| OPG005 | B22R | TLR signaling | Host-modulation |
| OPG016 |  |  | Host-modulation |
| OPG015 |  | Ankyrin | Host-modulation |
| OPG003 | C19L | Ankyrin | Host-modulation |
| OPG002 | C22L | TNF receptor | Host-modulation |
| OPG001 | B29R | Chemokine binding | Host-modulation |

This table summarizes the functional categories and descriptions of major mpox virus genes, as annotated in the reference genome NC_063383 and curated from ViralZone.

**Appendix 1p. A comprehensive workflow of genomic characterization of the mpox virus.**

**Appendix 2p. Intercontinental spread of MPOX during the 2022–2025 outbreak.**

Daily confirmed mpox cases per million population, based on World Health Organization (WHO) reports as of August 2025, adjusted to account for differences in regional population sizes. Red shading denotes South Korea, where a notable increase in case burden was observed following the rise in Japan, illustrating the temporal sequence of regional outbreaks.

**45**

**40**

**35**

**30**

**25**

**20**

**15**

**10**

**5**

**0**

**2022-04**

**2022-08**

**2022-12**

**2023-04**

**2023-08**

**2023-12**

**2024-04**

**2024-08**

**2024-12**

**2025-04**

**2025-08**

**Daily confirmed cases per million people**

**Date**

**Location**

**China**

**Democratic Republic of Congo**

**Europe**

**Japan**

**South Korea**

**United States**

**The first PHEIC(2022-2023), Global Ⅱb outbreak**

**The second PHEIC(2024-2025), Global Ⅰb outbreak**

**Onset of local transmission with case surge in South Korea**

**Appendix 3p. Overview of mpox virus genomes from South Korea: 110 newly generated sequences and 56 previously published sequences.**

| Sample ID | Nationality | Collection Date | Accession Number |
| --- | --- | --- | --- |
| MPXV-ROK-P023-2023 | South Korea | 2023-04-21 | PX379416 |
| MPXV-ROK-P025-2023 | China | 2023-04-22 | PX379417 |
| MPXV-ROK-P035-2023 | South Korea | 2023-04-26 | PX379418 |
| MPXV-ROK-P036-2023 | South Korea | 2023-04-26 | PX379419 |
| MPXV-ROK-P037-2023 | South Korea | 2023-04-26 | PX379420 |
| MPXV-ROK-P038-2023 | South Korea | 2023-04-26 | PX379421 |
| MPXV-ROK-P039-2023 | South Korea | 2023-04-27 | PX379422 |
| MPXV-ROK-P040-2023 | South Korea | 2023-04-27 | PX379423 |
| MPXV-ROK-P041-2023 | South Korea | 2023-04-27 | PX379424 |
| MPXV-ROK-P043-2023 | South Korea | 2023-04-28 | PX379425 |
| MPXV-ROK-P044-2023 | South Korea | 2023-04-29 | PX379426 |
| MPXV-ROK-P045-2023 | South Korea | 2023-04-30 | PX379427 |
| MPXV-ROK-P046-2023 | South Korea | 2023-04-30 | PX379428 |
| MPXV-ROK-P047-2023 | South Korea | 2023-04-30 | PX379429 |
| MPXV-ROK-P048-2023 | South Korea | 2023-05-01 | PX379430 |
| MPXV-ROK-P050-2023 | South Korea | 2023-05-02 | PX379431 |
| MPXV-ROK-P051-2023 | South Korea | 2023-05-02 | PX379432 |
| MPXV-ROK-P052-2023 | Philippine | 2023-05-02 | PX379433 |
| MPXV-ROK-P054-2023 | South Korea | 2023-05-03 | PX379434 |
| MPXV-ROK-P055-2023 | South Korea | 2023-05-04 | PX379435 |
| MPXV-ROK-P056-2023 | South Korea | 2023-05-04 | PX379436 |
| MPXV-ROK-P057-2023 | South Korea | 2023-05-03 | PX379437 |
| MPXV-ROK-P058-2023 | South Korea | 2023-05-04 | PX379438 |
| MPXV-ROK-P059-2023 | South Korea | 2023-05-04 | PX379439 |
| MPXV-ROK-P060-2023 | South Korea | 2023-05-05 | PX379440 |
| MPXV-ROK-P061-2023 | South Korea | 2023-05-08 | PX379441 |
| MPXV-ROK-P062-2023 | South Korea | 2023-05-08 | PX379442 |
| MPXV-ROK-P063-2023 | South Korea | 2023-05-08 | PX379443 |
| MPXV-ROK-P064-2023 | South Korea | 2023-05-08 | PX379444 |
| MPXV-ROK-P065-2023 | South Korea | 2023-05-09 | PX379445 |
| MPXV-ROK-P066-2023 | South Korea | 2023-05-09 | PX379446 |
| MPXV-ROK-P068-2023 | Taiwan | 2023-05-09 | PX379447 |
| MPXV-ROK-P069-2023 | South Korea | 2023-05-10 | PX379448 |
| MPXV-ROK-P071-2023 | South Korea | 2023-05-12 | PX379449 |
| MPXV-ROK-P072-2023 | South Korea | 2023-05-12 | PX379450 |
| MPXV-ROK-P073-2023 | Japan | 2023-05-12 | PX379451 |
| MPXV-ROK-P075-2023 | South Korea | 2023-05-13 | PX379452 |
| MPXV-ROK-P076-2023 | South Korea | 2023-05-16 | PX379453 |
| MPXV-ROK-P077-2023 | South Korea | 2023-05-17 | PX379454 |
| MPXV-ROK-P078-2023 | South Korea | 2023-05-17 | PX379455 |
| MPXV-ROK-P079-2023 | South Korea | 2023-05-17 | PX379456 |
| MPXV-ROK-P080-2023 | South Korea | 2023-05-18 | PX379457 |
| MPXV-ROK-P081-2023 | Russia | 2023-05-20 | PX379458 |
| MPXV-ROK-P082-2023 | South Korea | 2023-05-22 | PX379459 |
| MPXV-ROK-P083-2023 | South Korea | 2023-05-22 | PX379460 |
| MPXV-ROK-P084-2023 | South Korea | 2023-05-22 | PX379461 |
| MPXV-ROK-P085-2023 | South Korea | 2023-05-23 | PX379462 |
| MPXV-ROK-P086-2023 | South Korea | 2023-05-24 | PX379463 |
| MPXV-ROK-P087-2023 | Philippine | 2023-05-24 | PX379464 |
| MPXV-ROK-P088-2023 | South Korea | 2023-05-25 | PX379465 |
| MPXV-ROK-P089-2023 | South Korea | 2023-05-25 | PX379466 |
| MPXV-ROK-P091-2023 | South Korea | 2023-05-27 | PX379467 |
| MPXV-ROK-P096-2023 | South Korea | 2023-06-01 | PX379468 |
| MPXV-ROK-P105-2023 | China | 2023-06-07 | PX379469 |
| MPXV-ROK-P106-2023 | South Korea | 2023-06-09 | PX379470 |
| MPXV-ROK-P107-2023 | South Korea | 2023-06-12 | PX379471 |
| MPXV-ROK-P108-2023 | South Korea | 2023-06-14 | PX379472 |
| MPXV-ROK-P109-2023 | South Korea | 2023-06-15 | PX379473 |
| MPXV-ROK-P110-2023 | South Korea | 2023-06-15 | PX379474 |
| MPXV-ROK-P111-2023 | South Korea | 2023-06-16 | PX379475 |
| MPXV-ROK-P113-2023 | South Korea | 2023-06-19 | PX379476 |
| MPXV-ROK-P115-2023 | China | 2023-06-20 | PX379477 |
| MPXV-ROK-P118-2023 | South Korea | 2023-07-05 | PX379478 |
| MPXV-ROK-P119-2023 | South Korea | 2023-07-05 | PX379479 |
| MPXV-ROK-P120-2023 | South Korea | 2023-07-11 | PX379480 |
| MPXV-ROK-P121-2023 | South Korea | 2023-07-12 | PX379481 |
| MPXV-ROK-P122-2023 | South Korea | 2023-07-12 | PX379482 |
| MPXV-ROK-P123-2023 | South Korea | 2023-07-12 | PX379483 |
| MPXV-ROK-P126-2023 | South Korea | 2023-07-18 | PX379484 |
| MPXV-ROK-P128-2023 | South Korea | 2023-07-26 | PX379485 |
| MPXV-ROK-P129-2023 | South Korea | 2023-07-27 | PX379486 |
| MPXV-ROK-P131-2023 | South Korea | 2023-08-02 | PX379487 |
| MPXV-ROK-P132-2023 | South Korea | 2023-08-03 | PX379488 |
| MPXV-ROK-P133-2023 | South Korea | 2023-08-04 | PX379489 |
| MPXV-ROK-P134-2023 | South Korea | 2023-08-07 | PX379490 |
| MPXV-ROK-P136-2023 | South Korea | 2023-08-21 | PX379491 |
| MPXV-ROK-P137-2023 | South Korea | 2023-08-28 | PX379492 |
| MPXV-ROK-P138-2023 | South Korea | 2023-08-30 | PX379493 |
| MPXV-ROK-P139-2023 | Türkiye | 2023-09-04 | PX379494 |
| MPXV-ROK-P141-2023 | South Korea | 2023-09-05 | PX379495 |
| MPXV-ROK-P142-2023 | South Korea | 2023-09-13 | PX379496 |
| MPXV-ROK-P143-2023 | China | 2023-09-13 | PX379497 |
| MPXV-ROK-P144-2023 | South Korea | 2023-09-16 | PX379498 |
| MPXV-ROK-P145-2023 | South Korea | 2023-09-20 | PX379499 |
| MPXV-ROK-P147-2023 | South Korea | 2023-09-26 | PX379500 |
| MPXV-ROK-P148-2023 | South Korea | 2023-09-27 | PX379501 |
| MPXV-ROK-P149-2023 | South Korea | 2023-10-06 | PX379502 |
| MPXV-ROK-P150-2023 | South Korea | 2023-10-10 | PX379503 |
| MPXV-ROK-P151-2023 | South Korea | 2023-10-10 | PX379504 |
| MPXV-ROK-P152-2023 | South Korea | 2023-10-12 | PX379505 |
| MPXV-ROK-P154-2023 | South Korea | 2023-11-22 | PX379506 |
| MPXV-ROK-P156-2024 | South Korea | 2024-04-22 | PX379507 |
| MPXV-ROK-P158-2024 | South Korea | 2024-06-14 | PX379508 |
| MPXV-ROK-P159-2024 | South Korea | 2024-06-21 | PX379509 |
| MPXV-ROK-P160-2024 | South Korea | 2024-07-04 | PX379510 |
| MPXV-ROK-P161-2024 | South Korea | 2024-07-04 | PX379511 |
| MPXV-ROK-P162-2024 | South Korea | 2024-07-10 | PX379512 |
| MPXV-ROK-P163-2024 | South Korea | 2024-07-17 | PX379513 |
| MPXV-ROK-P165-2024 | South Korea | 2024-07-24 | PX379514 |
| MPXV-ROK-P167-2024 | South Korea | 2024-08-28 | PX379515 |
| MPXV-ROK-P168-2024 | South Korea | 2024-08-30 | PX379516 |
| MPXV-ROK-P173-2025 | South Korea | 2025-03-06 | PX379517 |
| MPXV-ROK-P174-2025 | South Korea | 2025-04-16 | PX379518 |
| MPXV-ROK-P175-2025 | South Korea | 2025-04-23 | PX379519 |
| MPXV-ROK-P176-2025 | South Korea | 2025-07-09 | PX379520 |
| MPXV-ROK-P100-2023 | South Korea | 2023-06-02 | PX379521 |
| MPXV-ROK-P103-2023 | South Korea | 2023-06-05 | PX379522 |
| MPXV-ROK-P157-2024 | South Korea | 2024-05-28 | PX379523 |
| MPXV-ROK-P164-2024 | South Korea | 2024-07-19 | PX379524 |
| MPXV-ROK-P166-2024 | South Korea | 2024-08-12 | PX379525 |
| MPXV-ROK-P1-2022 | South Korea | 2022-06-22 | OP204857.1 |
| MPXV-ROK-P002-2022 | South Korea | 2022-09-02 | OR459775 |
| MPXV-ROK-P003-2022 | South Korea | 2022-11-15 | OR459776 |
| MPXV-ROK-P004-2022 | South Korea | 2022-11-22 | OR459780 |
| MPXV-ROK-P005-2023 | South Korea | 2023-03-13 | OR459778 |
| MPXV-ROK-P006-2023 | South Korea | 2023-04-06 | OR459779 |
| MPXV-ROK-P007-2023 | South Korea | 2023-04-10 | OR459780 |
| MPXV-ROK-P008-2023 | South Korea | 2023-04-11 | OR459781 |
| MPXV-ROK-P009-2023 | South Korea | 2023-04-12 | OR459782 |
| MPXV-ROK-P010-2023 | South Korea | 2023-04-13 | OR459783 |
| MPXV-ROK-P011-2023 | South Korea | 2023-04-14 | OR459784 |
| MPXV-ROK-P012-2023 | South Korea | 2023-04-15 | OR459785 |
| MPXV-ROK-P013-2023 | South Korea | 2023-04-15 | OR459786 |
| MPXV-ROK-P014-2023 | South Korea | 2023-04-17 | OR459787 |
| MPXV-ROK-P015-2023 | South Korea | 2023-04-18 | OR459788 |
| MPXV-ROK-P016-2023 | South Korea | 2023-04-18 | OR459789 |
| MPXV-ROK-P017-2023 | South Korea | 2023-04-18 | OR459790 |
| MPXV-ROK-P018-2023 | South Korea | 2023-04-18 | OR459791 |
| MPXV-ROK-P019-2023 | South Korea | 2023-04-19 | OR459792 |
| MPXV-ROK-P020-2023 | South Korea | 2023-04-19 | OR459793 |
| MPXV-ROK-P021-2023 | South Korea | 2023-04-21 | OR459794 |
| MPXV-ROK-P022-2023 | South Korea | 2023-04-21 | OR459795 |
| MPXV-ROK-P024-2023 | South Korea | 2023-04-21 | OR459796 |
| MPXV-ROK-P026-2023- | South Korea | 2023-04-22 | OR459797 |
| MPXV-ROK-P027-2023 | South Korea | 2023-04-22 | OR459798 |
| MPXV-ROK-P028-2023 | South Korea | 2023-04-22 | OR459799 |
| MPXV-ROK-P029-2023 | South Korea | 2023-04-23 | OR459800 |
| MPXV-ROK-P030-2023 | South Korea | 2023-04-23 | OR459801 |
| MPXV-ROK-P032-2023 | South Korea | 2023-04-25 | OR459802 |
| MPXV-ROK-P033-2023 | South Korea | 2023-04-25 | OR459803 |
| MPXV-ROK-P034-2023 | South Korea | 2023-04-25 | OR459804 |
| MPXV-ROK-P053/01-2023 - MPXV-ROK-P053/24-2023 | South Korea | 2023-05-03 | OR855744 - OR855767 |
